# Supplementary material for: Considerations in implementation of social risk factor screening and referral in maternal and infant care in Washington, DC: A qualitative study
Source: PLoS One. 2023 Apr 13;18(4):e0283815. doi: 10.1371/journal.pone.0283815 (PMC10101493; doi:10.1371/journal.pone.0283815)
Supplement: S1 Table — (DOCX) [file pone.0283815.s001.docx]

| **Domain** | **OB/GYN** | **Pediatrics** | **Family Medicine** | **CBO** |
| --- | --- | --- | --- | --- |
| Food insecurity | Yes | Yes | Yes | Yes |
| Housing instability | Yes | Yes | Yes | Yes |
| Employment | Yes |  |  |  |
| Benefits (WIC/TANF/SNAP) | Yes | Yes | Yes |  |
| Baby supplies | Yes |  |  |  |
| Childcare | Yes |  |  |  |
| Legal help | Yes | Yes |  |  |
| Mental health | Yes | Yes | Yes | Yes |
| Education |  | Yes |  |  |
| Housing conditions (mold, bugs, mice, etc) | Yes | Yes |  | Yes |
| Utilities (electricity, heat, water) |  | Yes |  |  |
| Transportation to medical appointments | Yes |  |  | Yes |

**S1 Table. Domains covered in administered questionnaires**
